# Validation of the Chinese Manchester foot pain and disability index (C-MFPDI) among patients with inflammatory arthritis

**DOI:** 10.1186/s13047-019-0316-3

**Published:** 2019-01-23

**Authors:** Brina Xing Ying Erh, Hong-gu He, Kate Frances Carter, Peter P. Cheung, Daphne S. Tan, Wenru Wang, Keith Rome

**Affiliations:** 1 Alice Lee Centre for Nursing Studies, Yong Loo Lin School of Medicine, National University of Singapore, Level 2, Clinical Research Centre Bock MD11, 10 Medical Drive, Singapore, 117597 Singapore; 2 Division of Nursing, National University Hospital, Singapore, Singapore; 3 National University Health System, Singapore, Singapore; 4 Podiatry Department, Rehabilitation Centre, National University Hospital, Singapore, Singapore; 5 Podiatry Division, School of Health and Science, Western Sydney University, Sydney, Australia; 6 Department of Medicine, Yong Loo Lin School of Medicine, National University of Singapore, Singapore, Singapore; 7 Division of Rheumatology, National University Hospital, Singapore, Singapore; 8 Podiatry, Health and Rehabilitation Research Institute, Faculty of Health and Environmental Sciences, Auckland University of Technology, Auckland, New Zealand

**Keywords:** Foot, Chinese, Disability, Inflammatory arthritis, Pain, Psychometric, Singapore

## Abstract

**Background:**

The Manchester Foot Pain and Disability Index (MFPDI) is a patient-reported outcome tool used to measure foot pain and foot-related disability. The English version of the MFPDI has been successfully translated into other European languages, but there was no Chinese version to use in Chinese-speaking communities. The cross-sectional correlational study aimed to translate the MFPDI from English into simplified Chinese (C-MFPDI) and to test its psychometric properties among people with inflammatory arthritis in Singapore.

**Methods:**

The MFPDI was translated from English into Chinese using a forward-backward translation framework and was administered to 100 Chinese-speaking people with inflammatory arthritis. From the original 100 participants, 30 participants re-evaluated the C-MFPDI after 2 weeks. A Visual Analogue Scale and the Taiwan Chinese Foot Function Index in simplified Chinese were used to evaluate concurrent validity with the C-MFPDI. Health-related quality of life was assessed using the Chinese version of the European Quality of Life-5 Dimension to test construct validity.

**Results:**

The C-MFPDI had a high translation equivalent rate (96.3%) and content validity index (0.92), good internal consistency (Cronbach’s α = 0.90) and test-retest reliability (ICC = 0.87). The concurrent validity of the C-MFPDI was demonstrated to be acceptable through its significantly moderate to strong positive correlations with the Taiwan Chinese Foot Function Index (*r* = 0.62–0.72, *p* < 0.01) and Visual Analogue Scale foot pain (*r* = 0.65, *p* < 0.01). The C-MFPDI total scores were moderately negatively associated with Chinese European Quality of Life-5 Dimension utility scores (*r* = − 0.40, *p* < 0.01).

**Conclusion:**

The C-MFPDI had good psychometric properties. The C-MFPDI can be used to assess disabling foot pain, impairment and disability in Chinese-speaking people with inflammatory arthritis.

**Electronic supplementary material:**

The online version of this article (10.1186/s13047-019-0316-3) contains supplementary material, which is available to authorized users.

## Introduction

Foot pain, impairment and disability has been reported to be under-recognised in people with inflammatory arthritis (IA) that includes rheumatoid arthritis [1–4], psoriatic arthritis [5, 6], gout [7, 8], systemic lupus erythematous [9–11], spondyloarthritis [12, 13] and other forms of undifferentiated IA. Several generic and disease-specific foot scales have been developed to quantify the severity and impact of foot pain, impairment and disability within the context of rheumatic disease [14, 15] that includes the Foot Function Index [16], Leeds Foot Impact Scale [17], Foot Health Status Questionnaire [18] and the Manchester Foot Pain and Disability Index (MFPDI) [19]. The MFPDI is a self-administered questionnaire that assesses foot disability, pain and impairment [19]. It has been successfully translated from English into Danish, Spanish, Greek and Swedish [20–23] and validated in predominantly Caucasian populations [19, 24]. The MFPDI has been applied in an epidemiology-based study [25], clinical trial [26] and cross-sectional studies to assess the severity and impact of disabling foot pain in people with gout [7, 27] and early rheumatoid arthritis [28]. In clinical practice, it has also demonstrated utility in a multidisciplinary rheumatology foot clinic in a prominent hospital-based service in the UK [29]. Published studies suggest that the MFPDI is quick and easy to use [21, 29].

Foot problems in people with IA have been reported to be high in European populations but there is limited data in predominantly Chinese-speaking populations. In a hospital-based study foot pain, impairment and disability were highly prevalent in people with IA in Singapore [30]. However, Singapore’s majority population is Chinese and many older Singaporeans are not sufficiently proficient in the English language to enable questionnaire data to be collected without the aid of a translator [30]. The authors concluded that there was a need to translate a foot-specific patient-reported outcome measure into simplified Chinese to facilitate further research into IA-related foot pain in Asian communities. Therefore, the aims of this study were to translate the MFPDI from English into simplified Chinese and to test its psychometric properties among people with IA-related foot pain in Singapore.

## Methods

A cross-sectional correlational study design was evaluated in two stages. In stage one the original MFPDI version was translated into Chinese (C-MFPDI) (with items 18 and 19 removed) and stage 2 involved participants with IA completing the C-MFPDI and testing its psychometric properties. The 17-item MFPDI [19, 20, 22, 29] is divided into 3 domains: pain (5 items), functional limitation (10 items) and personal appearance (2 items). The total C-MFPDI score and the C-MFPDI subscale scores were calculated to: none of the time (score = 0), some days (score = 1) and on most day/every day(s) (score = 2). Ethical approval was granted by the ethics review board of the National Healthcare Group in Singapore (Domain Specific Review Board, 2015/00970). All participants provided written informed consent prior to data collection.

### Stage 1 translation of MFPDI and translational equivalence testing, pilot testing and content validity testing of the C-MFPDI

**Fig. 1:**
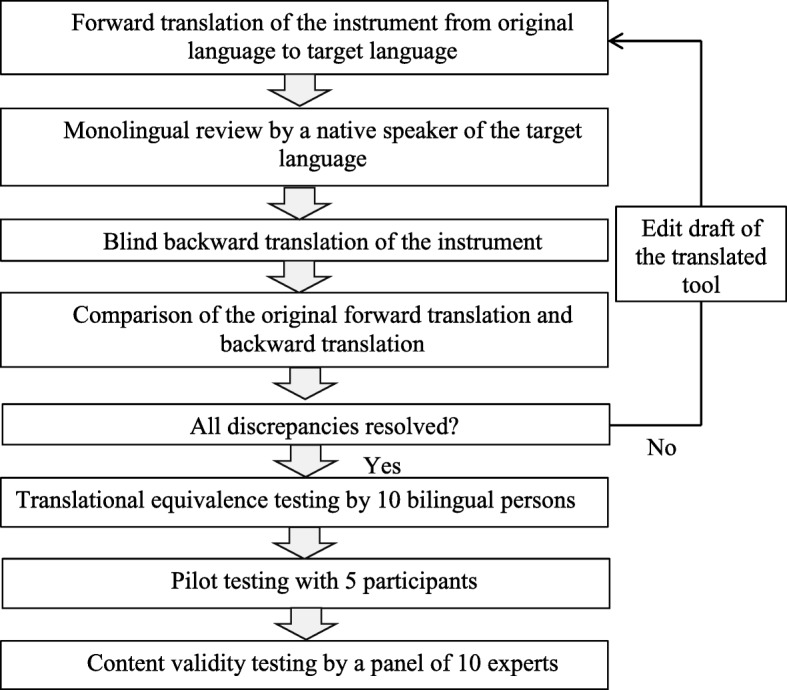
Forward-backward translation framework

Stage 1 was based on the back-translation model described by Brislin [31, 32] with reference to the translation guidelines by Oxford University Innovation (previously named Isis Innovation Ltd., UK). Copyright approval for use and hard-copy duplication of the instruments were obtained. The permission to translate the MFPDI into Chinese language and psychometric evaluation of the translated version was obtained from Oxford University Innovation. The translation protocol used is shown in Fig. 1. One bilingual researcher (BY) translated the MFPDI from English into simplified Chinese, which was then discussed and reviewed with two bilingual experts (HGH and WW) whose mother tongue was Chinese. The three forward translators are proficient in both spoken and written form of English and Chinese. As the three forward translators are familiar with healthcare terminologies and the Chinese culture, this approach helped to increase the accuracy of the translation and ensure that the wordings or phrases are appropriate for the local context [32, 33]. All amendments to the drafts of the C-MFPDI during the translation phase were made only after discussing and reaching common consensus amongst the three forward translators. The forward translation was followed by a monolingual review of the translated instrument by an invited native Chinese speaker, who is a student of the Master of Education (Chinese Language) at the National Institute of Education in Singapore, to enhance the accuracy and understanding of the C-MFPDI. The C-MFPDI draft was edited based on feedback from the monolingual review before proceeding with the back-translation. Subsequently, a blinded back-translation was performed by an independent bilingual translator without prior knowledge of the original MFPDI. The back-translator was an Honour’s student who was the first author’s classmate and she helped to reflect the actual meaning of the translated tool and assist in the clarification of words and phrases used during the translation process [33].

The team of three forward translators then compared and reviewed the original version, forward-translated version and backward-translated version of the MFPDI, focusing on eliminating ambiguous words, phrases and meanings [33]. Appropriate changes were made accordingly and the translation process was repeated until the original English MFPDI achieved a maximum equivalence with the back-translated English MFPDI.

Finally, translation equivalence testing was performed by 10 independent bilingual persons, who were colleagues, friends or relatives of the main researcher who were fluent in English and whose mother tongue is Chinese. They all held a minimum of GCE O Level’s pass qualification for both English and Chinese. They compared the original MFPDI against the final forward-translated version and evaluated the consistency of meaning between each item using a 4-point rating scale (“1” not equivalent, “2” somewhat equivalent, “3” equivalent, and “4” most equivalent). A translational equivalence index (TEI), the proportion of ratings as equivalent or most equivalent for each item, was calculated. A TEI of 0.90 or higher indicated a good translational equivalence [34].

Pilot testing was undertaken by 5 Chinese-speaking people with IA and known foot pain, who were recruited from the same study venue. They were invited to identify words, phrases or statements that were ambiguous or difficult to understand in the final translated questionnaire of the C-MFPDI. Content validity was conducted by an expert panel (two rheumatology consultants, two rheumatology nurses, three podiatrists and one nurse educator from hospital and academic institutions). Each item was rated according to its relevance to the underlying construct using a 4-point scale (“1” not relevant, “2” somewhat relevant, “3” relevant, and “4” very relevant). The item-content validity index (CVI) was calculated based on the proportion of “3” or “4” ratings. The scale-CVI was calculated by averaging across the 17 item-CVIs. A scale-CVI of 0.90 or higher indicated acceptable content validity of the C-MFPDI [35].

### Stage 2 psychometric properties testing of the C-MFPDI

A convenience sample of participants with rheumatologist-diagnosed IA (namely, Rheumatoid Arthritis, Psoriatic Arthritis, Spondyloarthritis, and undifferentiated Inflammatory Arthritis) were recruited from the rheumatology outpatient clinic of a tertiary public hospital in Singapore from December 2015 to March 2016. Participants were invited to complete the C-MFPDI after a 2-week interval. Participants were eligible if they were 21 years old and above (since they are considered as adults in Singapore and they can give independent consent to participate in the study), able to speak Mandarin Chinese and had experienced foot pain during the past month. Those with visual, speech and/or hearing difficulties, cognitive impairment, a current traumatic foot injury and/or those unable to walk 10 m unaided were excluded. The target sample size of the stage 2 was based on the required number of participants for psychometric evaluation of the 17-item C-MFPDI. Psychometric theories recommend five to 10 participants per item for psychometric evaluation of a questionnaire [36] while other researchers suggested a minimum of 100 participants to perform correlational and factor analysis [37]. A sample size target of minimally 100 participants was established. Permission from the participants in the primary group was sought before they were invited to complete the C-MFPDI again 2 weeks [38] after the first time data collection to establish the test-retest reliability. A copy of the C-MFPDI was passed to agreeable participants with a return envelope and a postage stamp. The participants were asked to mail it back to the co-investigator (HHG) after they completed the C-MFPDI 2 weeks later.

Demographic and clinical characteristics included age, gender, education level, employment status, the type of IA and disease duration. The tender and swollen joint count score (28-joints), erythrocyte sedimentation rate (ESR), C-reactive protein (CRP) and the physician global health assessment value were recorded to evaluate disease activity levels. The Disease Activity Score in 28-joints using ESR (DAS28-ESR) and CRP (DAS28-CRP) were calculated [39].

To evaluate concurrent validity, the C-MFPDI was compared to foot pain severity using a 100 mm Visual Analogue Scale (VAS) for ‘today’ and ‘the past month’ and the Taiwan Chinese Foot Function Index (FFI) [40] (modified into simplified Chinese). Each item of the Taiwan Chinese FFI is answered on a VAS according to experiences over the past week, higher scores indicate greater foot functional impairment [40]. The Taiwan Chinese FFI has been shown to have good internal consistency, test-retest reliability and criterion validity [40]. The Taiwan Chinese FFI subscale scores (for foot-related pain, disability and activity limitation) were calculated by totalling the scores of all applicable items within a subscale and dividing the total number of applicable items [40]. To evaluate construct validity, the disability domain of the C-MFPDI was compared to the Chinese version of the European Quality of Life-5 Dimension (EQ-5D) [41, 42]. The approval of using the TW-FFI and EQ-5D was obtained.

### Statistical analysis

All statistical tests were conducted using SPSS Version 23.0 (IBM Corp., Armonk, NY). Demographic, clinical characteristics, the TEI and CVI were described using descriptive statistics. The reliability of the C-MFPDI was determined by internal consistency and test-retest reliability. Internal consistency was evaluated using item-to-total correlation coefficient and Cronbach’s α test [35, 43]. An item-to-total correlation coefficient of 0.4 and above is desirable and shows that the item of concern is consistent with the behaviour of the other items [43]. The Cronbach’s α coefficient ranges from 0.00 to 1.00 where the higher the Cronbach’s α value, the higher the internal consistency reliability [35]. Test-retest reliability was evaluated using the intra-class correlation coefficient (ICC) [35]. An ICC > 0.70 would be considered adequate while a value of > 0.80 would be highly desirable [35].

Various strategies were used to examine the validity of the C-MFPDI. The content validity was tested by calculating the scale-CVI, where a value of 0.90 or higher indicates satisfactory content validity [35]. The concurrent validity of the C-MFPDI was examined using the Pearson product-moment correlation coefficient by testing the relationship between the C-MFPDI scores and VAS scores as well as TW-FFI scores [35]. Coefficients of 0.70 or higher indicate strong correlation while coefficients from 0.3 to 0.7 indicate moderate correlation [35]. The construct validity of the C-MFPDI was tested by the hypothesis testing approach through examining the relationship between the disability domain of the C-MFPDI and the Chinese version of the EQ-5D using the Pearson product-moment correlation coefficient [35]. It was hypothesized a priori that a negative relation would be found between disabling foot pain measured using the C-MFPDI and HRQoL measured by the EQ-5D. Principal components factor analysis (exploratory factor analysis) with varimax rotation was finally used to identify the underlying structure of the C-MFPDI items [35]. A *p*-value of less than 0.05 was consisted statistical significant.

The Kolmogorov-Smirnov test, skewness, kurtosis and the normal Quantile-Quantile (Q-Q) plots were used to check the normality of the data [44, 45]. The results showed that all outcome variables had *p* < 0.05 [44]. The skewness and kurtosis for all variables were within + 1 and − 1 range except for VAS pain scale for ‘today’ and the three subscales for the TW-FFI. However, the normal QQ plots for all outcome variables revealed that all points laid in a random scatter around the lines. With reference to the central limit theorem, if the sample has a large sample size (*n* > 30), the sampling distribution will tend to have a normal distribution regardless of the shape of the data [45]. Thus parametric tests were used in this study.

## Results

### Stage 1: Translational equivalence testing, pilot testing and content validity testing results

Ten bilingual persons completed and returned the translational equivalence testing forms. Items 2, 8, and 14 scored item-TEIs of 0.8; item 9 scored an item-TEI of 0.9; and the remaining items scored item-TEIs of 1.0. Revisions were made to items rated of TEI less than 0.9 based on the bilingual authors’ feedback and sent back to the 10 bilingual persons for evaluating. Eventually, the overall TEI of the C-MFPDI was 0.96 suggesting good translational equivalence.

The pilot testing involved five participants with IA and foot pain, who completed and returned the feedback form and gave verbal feedback immediately after completion. All pilot participants were able to complete the C-MFPDI in 5 min independently and agreed unanimously that the response options in the C-MFPDI were relevant to them. One pilot participant felt that the C-MFPDI was “generally easy to understand, difficult to answer if it did not apply to me now”. All pilot participants but one did not express difficulty understanding the C-MFPDI. The one participant informed that the phrases and sentences took rather long to comprehend and would have been better if they were “more relatable to Singaporean Chinese”. The team of forward translators looked through the comments together and did not make any changes to the C-MFPDI draft during this stage to keep the meaning of items in the C-MFPDI as close to the meaning of items in the original MFPDI as possible. The local researcher had gone through the items phrasing again and found no difficulty in understanding the instrument from a native Singaporean Chinese’s point of view. The results from the pilot testing stage showed that the C-MFPDI is generally quick and easy to comprehend and complete.

Eight experts were involved in the content validity testing. Item 6, item 8, and item 11 scored item-CVIs of 0.72. Item 12 and item 15 scored item-CVIs of 0.86. The remaining items scored item-CVIs of 1. The Scale-CVI of the C-MFPDI was 0.92. Revisions were made to items with a CVI of less than 0.80 based on the comments by the experts. A final version of the C-MFPDI was locked down after concluding edits (Additional file 1).

After going through the aforementioned process, the original item 8 ‘I catch the bus or use the car more often’ was translated to ‘I take public transport or use the car more often’ in Chinese mandarin language, taking into account the differences in Singapore’s and UK’s transport patterns. Extra attention was paid to items 6, 11, 12, 15 and 17. Item 6 was considered not very relevant as the walking paths in Singapore are generally smooth. For items 11 and 12, it was difficult to translate the concept of “self-conscious” into Chinese mandarin language. There is no Chinese phrase that could contain the meaning of having undue awareness of oneself or one’s appearance. After discussion amongst the three translators, the Chinese phrase that meant to ‘take notice of’ was selected as the final wording to represent ‘feel self-conscious about’ in the C-MFPDI. For the item 15, one expert thought the way of phrasing the sentence should be similar with item 16 to improve the understanding. Similarly, there is also no equivalent phrasing of ‘shooting pains’ of item 17 in the Chinese mandarin language, the Chinese phrasing that meant ‘severe and sharp pain’ was selected for the final translation. Items 18 “Because of pain in my feet, I am unable to carry out my previous work” and 19 “Because of pain in my feet, I no longer do all my previous activities (sport, dancing, hill walking etc)” were not included in the C-MFPDI.

### Stage 2 psychometric properties testing results

A total of 132 participants were approached between December 2015 and March 2016 until 100 subjects were recruited. A total of 32 (24%) patients were not included, as they did not fall within the eligibility criteria or refused participation stating they were rushed for time or already participating in on-going and previous studies and citing personal reasons such as feeling unwell. A total of 49 participants were invited to complete the C-MFPDI again at 2 weeks after the first questionnaire-survey until 30 responses were received.

One hundred participants completed the C-MFPDI (Additional file 1 for the translated instrument). Demographic and clinical characteristics are shown in Table 1. Most participants were female (67%, *n* = 67) who had received a secondary education and tertiary education (71%, *n* = 71). The mean (SD) age was 52.9 (14.7) years and 22 (22%) participants reported use of an assistive device for walking. The most common IA condition was Rheumatoid arthritis (70%, *n* = 70) followed by Spondyloarthritis (17%, *n* = 17). The mean (SD) disease duration was 7.9 (8.6) years. The DAS28-ESR indicated moderate levels of disease activity with a mean (SD) score of 3.4 (1.1). Table 2 demonstrates the mean (SD) scores for the C-MFPDI, Taiwan Chinese FFI, VAS for foot pain and the Chinese version of the EQ-5D.

**Table 1:** Demographic and clinical characteristics (*n* = 100)

Demographic and clinical characteristics	Mean (SD)	n (%)
Female		67 (67%)
Age (Years)	52.9 (14.7)	
Educational level		
Tertiary (‘A’ levels, Diploma, Degree)		41 (41%)
Employment status		
Full-time		44 (44%)
Retired		20 (20%)
Part time/ Schooling/Homemaker		26 (26%)
Unemployed due to IA symptoms/other reasons		10 (10%)
Diagnosis and duration since diagnosis	7.9 (8.6)	
Rheumatoid arthritis	8.6 (8.9)	70 (70%)
Psoriatic arthritis	5.8 (5.8)	5 (5%)
Spondyloarthritis	12.2 (12.6)	17 (17%)
Undifferentiated inflammatory arthritis	7.7 (8.6)	8 (8%)
IA symptoms impacted daily activities (Yes)		71 (71%)
IA symptoms impacted social activities (Yes)		28 (28%)
IA symptoms impacted work ability (Yes)		32 (32%)
Ever visited podiatrist (Yes)		29 (29%)
Use of assistive device for walking (Yes)		22 (22%)
Tender joint count (28)	2.6 (4.4)	
Swollen joint count (28)	1.0 (2.4)	
DAS28-ESR^b^ score (*n* = 74)	3.4 (1.1)	
DAS28-CRP^b^ score (*n* = 24)	3.0 (0.9)	
DAS28-ESR^b^ score ranking (n = 74)		
Disease in remission (DAS28 < 2.6)		17 (23%)
Low disease activity (DAS28 > 2.6 but</=3.2)		24 (32%)
Moderate Disease activity (DAS28 > 3.2 but< 5.1)		28 (38%)
Active disease (DAS28 > 5.1)		5 (7%)

^a^ Missing data: *n* = 10; ^b^ Only for patients with Rheumatoid arthritis

**Table 2:** Mean (SD) outcome variable scores (*n* = 100)

Outcomes	Mean (SD)	Actual range	Possible range
VAS^a^ for foot pain (today)	1.6 (2.3)	0–8	0–10
VAS for foot pain (month)	2.9 (2.6)	0–8	0–10
Taiwan Chinese FFI^b^ pain	1.8 (2.1)	0–8	0–10
Taiwan Chinese FFI disability	1.9 (2.4)	0–9.7	0–10
Taiwan Chinese FFI activity limitation	1.4 (2.1)	0–8.7	0–10
C-MFPDI^c^ pain intensity	2.0 (2.3)	0–8	0–10
C-MFPDI personal appearance	1.3 (1.5)	0–4	0–4
C-MFPDI functional limitation	4.6 (5.1)	0–20	0–20
C-MFPDI total score	7.8 (7.5)	0–30	0–34
EQ-5D^d^ utility score	0.7 (0.3)	−0.25 - 1.00	− 0.77 - 1.00
EQ-5D global-VAS	67.5 (17.9)	20 - 100	0–100

^a^Visual Analogue Scale; ^b^ Foot Function Index; ^c^ Chinese Manchester Foot Pain and Disability Index; ^d^ European Quality of Life - 5 Dimensions - 3 Levels

As shown in Table 3, the internal consistency was excellent for the C-MFPDI (Cronbach’s α =0.90) for the total scale, with the subscale Cronbach’s α values ranged from 0.70 to 0.90. Test-retest reliability (*n* = 30) had an ICC of 0.76–0.87 for the subscale scores and 0.87 for the total score (Table 3). Item-to-total correlation ranged between 0.34 and 0.73 (Table 3). Moderate to strong positive correlations were found between the C-MFPDI total scores and VAS foot pain scores on day of administration (*r* = 0.48, *p* < 0.01), VAS foot pain for the past month (*r* = 0.65, *p* < 0.01), as well as TW-FFI pain scores (r = 0.72, *p* < 0.01), TW-FFI disability scores (r = 0.70, *p* < 0.01), and TW-FFI activity limitation scores (r = 0.62, *p* < 0.01). When examining the correlations among subscale scores of VAS, C-MFPDI and TW-FFI, moderate positive correlations were found between the C-MFPDI functional limitation subscale scores and the Taiwan Chinese FFI disability scale scores (r = 0.68, *p* < 0.01) as well as Taiwan Chinese FFI activity limitation scale scores (r = 0.61, *p* < 0.01) (Table 4). Strong or moderate significantly positive correlations were found between the C-MFPDI pain intensity scores and TW-FFI pain scores (*r* = 0.74, *p* < 0.01), as well as VAS foot pain scores on day of administration (*r* = 0.65, *p* < 0.01) and for the past month (*r* = 0.65, *p* < 0.01). The C-MFPDI total score was moderately negatively associated with the Chinese version of the EQ-5D utility scores (r = − 0.40, *p* < 0.01).

**Table 3:** Internal consistency and test-retest reliability of the C-MFPDI

Constructs	Items of the C-MFPDI^a^	Item-to-total correlation (*n* = 100)	Cronbach’s α (*n* = 100)	Intra-class coefficient (*n* = 30)
Physical limitation			0.90	0.87
	Item 1	0.67		
	Item 2	0.70		
	Item 3	0.66		
	Item 4	0.72		
	Item 5	0.71		
	Item 6	0.56		
	Item 7	0.73		
	Item 8	0.68		
	Item 9	0.59		
	Item 10	0.61		
Personal appearance			0.71	0.82
	Item 11	0.55		
	Item 12	0.55		
Pain intensity			0.70	0.76
	Item 13	0.47		
	Item 14	0.54		
	Item 15	0.39		
	Item 16	0.34		
	Item 17	0.59		
Total score			0.90	0.87

Note: ^a^Simplified Chinese version of the Manchester Foot Pain and Disability Index

**Table 4:** Correlations of Outcome variables (*n* = 100)

Correlations	1 ^a^	2^b^	3^c^	4^d^	5^e^	6^f^	7^g^	8^h^	9^i^	10^j^	11^k^
1. VAS (today) ^a^	1										
2. VAS (month)^b^	0.58^**^	1									
3. C-MFPDI-FL^c^	0.39^**^	0.62^**^	1								
4. C-MFPDI-PA^d^	0.15^**^	0.19	0.40^**^	1							
5. C-MFPDI-PI^e^	0.65^**^	0.65^**^	0.68^**^	0.35^**^	1						
6. C-MFPDI total^f^	0.48^**^	0.65^**^	0.96^**^	0.57^**^	0.82^**^	1					
7. TW-FFI-P^g^	0.60^**^	0.76^**^	0.67^**^	0.24^*^	0.74^**^	0.72^**^	1				
8. TW-FFI-D^h^	0.49^**^	0.63^**^	0.68^**^	0.21^*^	0.66^**^	0.70^**^	0.82^**^	1			
9. TW-FFI -AL^i^	0.37^**^	0.41^**^	0.61^**^	0.26^**^	0.54^**^	0.62^**^	0.60^**^	0.71^**^	1		
10. EQ-5D utility score^j^	−0.24^*^	−0.21^*^	−0.42^**^	−0.07	−0.35^**^	−0.40^**^	−0.30^**^	−0.43^**^	−0.49^**^	1	
11. EQ-5D G-VAS^k^	−0.19	−0.20^*^	−0.30^**^	−0.06	−0.28^**^	−0.30^**^	−0.17	−0.23^*^	−0.24^*^	0.36^**^	1

* *p* < 0.05 (2-tailed)** *p* < 0.01 (2-tailed); ^a^ Visual analogue scale for foot pain on the day of survey; ^b^ Visual analogue scale for foot pain for the past month; ^c^ C-MFPDI functional limitation subscale score; ^d^ C-MFPDI personal appearance subscale score; ^e^ C-MFPDI pain intensity subscale score; ^f^ C-MFPDI total score; ^g^ Taiwan Chinese FFI pain subscale score; ^h^ Taiwan Chinese FFI disability subscale score; ^I^ Taiwan Chinese FFI activity limitation subscale score; ^j^ European Quality of Life – 5 Dimensions – 3 Levels utility score; ^k^ European Quality of Life – 5 dimensions – global visual analogue scale

## Discussion

The study to the author’s knowledge is the first to translate an instrument that measures disabling foot pain into simplified Chinese. The study found that the C-MFPDI produced good psychometric properties. During the translation process a few translational problems were encountered. We found due to the different transport patterns between Singapore and the UK, item 8 was not translated literally in view of lifestyle differences. In Singapore, buses, railway and taxis are the means of public transport [46]. However, in the UK, the railway is generally taken for long distance traveling and urban railway networks are only available in major cities. On the other hand, bus services cover the whole of UK [47] and the original item 8 could have been phrased to enhance its generalizability across the whole of UK. Therefore, the original item ‘I catch the bus or use the car more often’ was translated to ‘I take public transport or use the car more often’ in Chinese mandarin language, taking into account the differences in Singapore’s and UK’s transport patterns. In addition, it was difficult to translate into Mandarin Chinese the concept of “self-conscious”, which means undue awareness of oneself or one’s appearance. This difficultly of expressing a concept in a language other than its original language had also been faced in the translation studies of other instruments [20, 48, 49]. The Chinese phrase meaning “take notice of” was selected to represent “feel self-conscious about” in the C-MFPDI. In addition, there was no equivalent word for the phrase of “shooting pains” in item 17 in Chinese. While the perception of “shooting pain” may be different from person to person, it covers the experience of having “sudden sharp and severe pain”, which was then selected for the final translation. Similar difficulties have previously been documented with the Greek translation of the MFPDI [22]. Furthermore, several experts thought that the question relating to the surfaces of walking paths (item 6) was not particularly relevant to the local context as the walking paths in Singapore are generally smooth. A general problem faced when translating patient reported outcomes are that language concepts are never exactly equivalent, affected by unique cultural contexts. Item 18 and 19 were not included in the C-MFPDI as these items tend to be non-applicable when patients are of retirement age, which was a great proportion of patients with foot pain according to previous studies [20, 22].

To better target and treat inflammation present in the foot it is important that the extent and impact of this inflammation are identified, alongside reliable and valid patient-reported outcomes. The C-MFPDI could be used in daily clinical practice and research as a way to quantify a patient’s perception of foot pain, disability and impairment. Foot-specific measures such as the MFPDI have been advocated amongst podiatrists to inform escalation or tapering of care based on monitoring foot disease [14, 50]. The routine use of the C-MFPDI in our multidisciplinary rheumatology clinic in Singapore has helped to identify the foot health needs of this patient group, prompt referral of patients to targeted health professionals and to inform treatment targets.

There are a number of limitations to the study. Convenience sampling was used to recruit participants from a multidisciplinary rheumatology outpatient clinic in a tertiary hospital based in Singapore and thus the findings may not be generalizable to the broader Chinese-speaking community. Future cross-cultural studies are indicated to evaluate the utility of the C-MFPDI among other Chinese-speaking populations worldwide. The study recruited from a mixed rheumatology caseload and grouped different IA conditions together for analysis, whereas previous studies using the MFPDI have focused on a single condition [7, 27, 28]. Although a mixed IA caseload provides a true representation of clinical practice, using a heterogeneous cohort potentially limits study comparison and generalizability of findings. The last two items of the MFPDI (item 18 and 19) that related to the difficulty in performing work or leisure activities were excluded from the questionnaire in the present study. Whilst these items have been excluded in other published studies if the respondent was of retirement age [20, 22, 29], only 20% (*n* = 20) of our study sample were retired. This should be accounted for in future research. Participants were recruited from a multidisciplinary rheumatology clinic and considered to be receiving optimal pharmacological and non-pharmacological management. This could have resulted in lower levels of global and/or local disease activity. However, cultural variations may also affect foot disease in IA and further research is warranted. Patient self-report may also have under-estimated foot problems and inclusion of a clinical foot assessment in future work is indicated. The MFPDI is a patient reported outcome measure developed and validated to measure pain specifically related to a foot disability. Quantifying foot pathologies is important, however, the aim of the study was to translate the MFPDI from English into simplified Chinese (C-MFPDI) and to test its psychometric properties among people with inflammatory arthritis in Singapore. Lastly, several psychometric measures for responsiveness to change, and floor to ceiling effect were not included in the study.

## Conclusion

The findings indicate that the simplified Chinese language version of the MFPDI is a valid and reliable tool in measuring foot pain, impairment and disability among Chinese-speaking people based in Singapore with inflammatory arthritis. Further research using the C-MFPDI will facilitate a better understanding of inflammatory arthritis foot involvement in Asian communities and will provide opportunity for wider international collaboration and comparison between populations.

## Additional file

Additional file 1: The Chinese version of the MFPDI. (DOCX 17 kb)

## Abbreviations

C-MFPDI: Simplified Chinese version of the Manchester foot pain disability index
CRP: C-reactive protein
CVI: Content validity index
DAS28-CRP: Disease activity score in 28-joints using CRP
DAS28-ESR: Disease activity score in 28-joints using ESR
EQ-5D: European Quality of Life-5 Dimension
ESR: Erythrocyte sedimentation rate
FFI: Foot Function Index
IA: Inflammatory Arthritis
ICC: Intra-class correlation coefficient
MFPDI: Manchester Foot Pain and Disability Index
TEI: Translational equivalence index
VAS: Visual Analogue Scale
