# Supplementary material for: Validation of the Chinese Manchester foot pain and disability index (C-MFPDI) among patients with inflammatory arthritis
Source: J Foot Ankle Res. 2019 Jan 23;12:6. doi: 10.1186/s13047-019-0316-3 (PMC6343242; doi:10.1186/s13047-019-0316-3)
Supplement: Supplementary file 1 — The Chinese version of the MFPDI. (DOCX 17 kb) [file 13047_2019_316_MOESM1_ESM.docx]

**Supplementary File 1.** The Chinese version of the MFPDI.

**曼切斯特足部疼痛及残疾指数-简体中文版 (C-MFPDI)**

以下是一些关于人们因为足部疼痛所面临问题的陈述。

对于下列每一项陈述，请指出它是否符合您在过去一个月内的经历。若符合，这种情况是发生在过去一个月中的某些日子还是大多数的日子/每天？

| **请在每个符合您陈述的选项内打勾。** | **在过去一个月内我有这个经历：** | | | |  |
| --- | --- | --- | --- | --- | --- |
| **因为我的足部疼痛：** | 任何时候都没有 | 某些日子 | 大多数日子/每天 | |  |
| 我完全避免在外面行走 |  |  |  | |  |
| 我避免走远路 |  |  |  | |  |
| 我无法正常地行走 |  |  |  | |  |
| 我慢慢地行走 |  |  |  | |  |
| 我得停下来歇脚 |  |  |  | |  |
| 我尽量避免在坚硬或粗糙的路面上走 |  |  |  | |  |
| 我避免长时间站立 |  |  |  | |  |
| 我更常乘搭公共交通工具或使用汽车 |  |  |  | |  |
| 我需要（别人）帮助做家务/购物 |  |  |  | |  |
| 足部疼痛时我变得烦躁 |  |  |  | |  |
| 我在任何情况都会很在意足部 |  |  |  | |  |
| 我会很在意该穿什么样的鞋子 |  |  |  | |  |
| 我依然做所有的事情，但是会有更多的疼痛和不适 |  |  |  | |  |
| 我有持续性的足部疼痛 |  |  |  | |  |
| 我的足部（情况）在早晨时更差 |  |  |  | |  |
| 我的足部在晚上时更加疼痛 |  |  |  | |  |
| 我的足部会有剧烈刺痛 |  |  |  | |  |
| **请您在读完上述每一条陈述后， 在这里打一个勾** | | | |  | |
